# A Review of Serotonin in the Developing Lung and Neonatal Pulmonary Hypertension

**DOI:** 10.3390/biomedicines11113049

**Published:** 2023-11-14

**Authors:** Jamie L. Archambault, Cassidy A. Delaney

**Affiliations:** Section of Neonatology, Department of Pediatrics, University of Colorado, Aurora, CO 80045, USA; jamie.archambault@childrenscolorado.org

**Keywords:** serotonin, pulmonary hypertension, neonate

## Abstract

Serotonin (5-HT) is a bioamine that has been implicated in the pathogenesis of pulmonary hypertension (PH). The lung serves as an important site of 5-HT synthesis, uptake, and metabolism with signaling primarily regulated by tryptophan hydroxylase (TPH), the 5-HT transporter (SERT), and numerous unique 5-HT receptors. The 5-HT hypothesis of PH was first proposed in the 1960s and, since that time, preclinical and clinical studies have worked to elucidate the role of 5-HT in adult PH. Over the past several decades, accumulating evidence from both clinical and preclinical studies has suggested that the 5-HT signaling pathway may play an important role in neonatal cardiopulmonary transition and the development of PH in newborns. The expression of TPH, SERT, and the 5-HT receptors is developmentally regulated, with alterations resulting in pulmonary vasoconstriction and pulmonary vascular remodeling. However, much remains unknown about the role of 5-HT in the developing and newborn lung. The purpose of this review is to discuss the implications of 5-HT on fetal and neonatal pulmonary circulation and summarize the existing preclinical and clinical literature on 5-HT in neonatal PH.

## 1. Introduction

Serotonin (5-HT) is a bioamine most appreciated for its role as a neurotransmitter and its effect on mood and behavior. However, the majority of 5-HT is synthesized in the periphery where 5-HT modulates numerous other biologic processes including respiratory, cardiovascular, and gastrointestinal functions [1,2,3]. Over the past several decades, 5-HT has emerged as a potent pulmonary vasoconstrictor and angiogenic agent that plays an important role in the development of pulmonary hypertension (PH).

**Figure 1:**
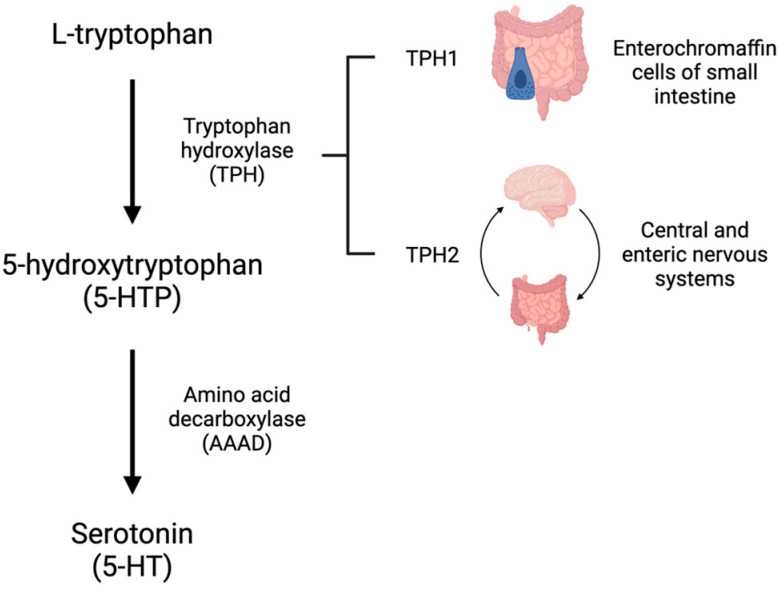
Serotonin (5-HT) is synthesized from the amino acid tryptophan. L-tryptophan is converted to 5-hydroxytryptophan (5-HTP) by tryptophan hydroxylase (TPH). TPH1 exists in the enterochromaffin cells of the small intestine and TPH2 is primarily found in the central and enteric nervous systems. 5-HTP is converted to 5-HT by amino acid decarboxylase (AAAD).

**Figure 2:**
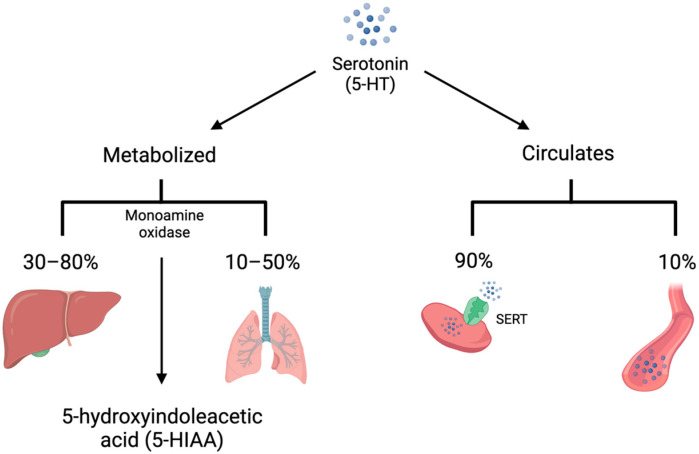
Serotonin (5-HT) is either metabolized by monoamine oxidase to 5-hydroxyindoleacetic acid (5-HIAA) or circulates in the bloodstream. The majority of 5-HT is metabolized on first pass by the liver, with much of the remainder metabolized by the lungs. In addition, 90% of unmetabolized 5-HT is taken up into platelets via the 5-HT transporter (SERT). Only 10% of circulating 5-HT is free in the plasma.

5-HT is synthesized from L-tryptophan through the activity of tryptophan hydroxylase (TPH), the rate-limiting enzyme in 5-HT synthesis. TPH has two distinct isoforms. TPH1 is primarily present in the enterochromaffin cells of the small intestine and is responsible for the production of peripheral 5-HT, whereas TPH2 is present in the central and enteric nervous systems [4,5]. TPH converts L-tryptophan to 5-hydroxytroptophan (5-HTP). 5-HTP is then converted by amino acid decarboxylase (AAAD) to 5-HT (Figure 1). On first pass, 30–80% of 5-HT synthesized in the intestine is metabolized by the liver and 90% of the remainder is metabolized by the lungs. The remaining 10% is primarily stored in platelets which take up 5-HT via the 5-HT transporter (SERT) (Figure 2). Consequently, only a small amount of 5-HT freely circulates and plasma levels are extremely low [6].

The diverse biologic functions of 5-HT are mediated by fifteen unique receptors divided into seven families (5-HT 1–7) based on molecular structure, signal transduction properties, and pharmacological properties [7,8]. The 5-HT 1, 2, and 4–7 receptors couple with G-proteins, while the 5-HT 3 receptors are 5-HT-gated ion channels. The altered expression and activity of both SERT and 5-HT receptors have been implicated in the pathophysiology of a wide spectrum of conditions in the fetus, newborns, and adults. This review aims to describe the role of pulmonary 5-HT signaling, discuss its implications on the adult lung, and summarize the existing preclinical and clinical literature investigating the role of 5-HT in the developing lung and neonatal PH.

## 2. Serotonin in Adult Pulmonary Circulation

### 2.1. Serotonin Signaling in the Adult Lung

**Figure 3:**
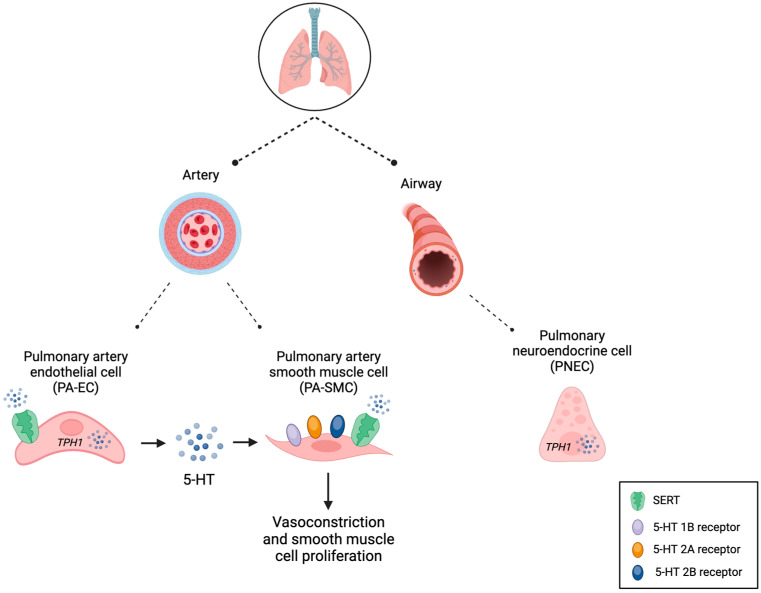
The lung participates in serotonin (5-HT) synthesis, uptake, and metabolism. 5-HT is synthesized by pulmonary artery endothelial cells (PA-EC) and pulmonary neuroendocrine cells (PNEC). 5-HT is taken up via SERT by both endothelial and smooth muscle cells of the pulmonary arteries. Pulmonary artery smooth muscle cells (PA-SMC) also express G-protein-coupled receptors which, upon binding 5-HT, contribute to vasoconstriction and smooth muscle cell proliferation seen in pulmonary hypertension (PH).

The lung is an important site of 5-HT synthesis, uptake, and metabolism (Figure 3). Pulmonary artery endothelial cells (PA-EC) that line the intima of the pulmonary vasculature, along with pulmonary neuroendocrine cells (PNEC) that line the airway epithelium, express TPH1 and contribute to local 5-HT synthesis. Preclinical studies have demonstrated that PA-EC synthesis of TPH1 is upregulated under hypoxic conditions, leading to increased local production of 5-HT [9,10]. The lung also plays an important role in the clearance of 5-HT via SERT-mediated uptake by PA-ECs, primarily at the site of pulmonary arterioles and capillaries [11,12,13,14]. The PA-EC uptake of 5-HT is a saturable, energy-dependent process that serves as the rate-limiting step in pulmonary clearance of circulating 5-HT [12]. Once taken up by PA-ECs, 5-HT is converted into its active metabolite 5-hydroxyindolacetic acid (5-HIAA) by the intracellular mitochondrial enzyme monoamine oxidase (MAO) [15,16].

SERT and the 5-HT G-protein-coupled receptors 1B, 2A, and 2B expressed by pulmonary artery smooth muscle cells (PA-SMC) serve to mediate 5-HT-induced vasoconstriction and pulmonary vascular remodeling [17,18,19,20,21]. 5-HT intracellular signaling is complex and binding to these receptors stimulates the induction of reactive oxygen species, activation of Rho-kinase, and mitogen activation of the protein kinase pathway [22,23]. Phosphorylated kinases travel to the nucleus to increase the transcription of growth factors, promote cellular proliferation, and contribute to vasoconstriction [24]. In non-human mammals the 5-HT 2A receptor mediates pulmonary vasoconstriction, while the 5-HT 1B receptor mediates both pulmonary vasoconstriction and PA-SMC proliferation [18,20,25]. When aberrantly regulated, these processes significantly contribute to the pathophysiology underlying PH

### 2.2. Serotonin in Adult Pulmonary Hypertension

The 5-HT hypothesis of PH was first proposed in the 1960s following the observation that the use of appetite suppressants such as aminorex, fenfluramine, and chlorphentermine led to the development of PH [26,27,28,29,30,31,32]. These drugs are known to increase extracellular levels of 5-HT in both the brain and the lung by exchanging exogenous drug molecules for endogenous 5-HT, thus providing substrate for 5-HT uptake/transport and contributing to the development of PH. This association was further supported by the observation that patients with altered platelet 5-HT storage develop PH [33,34,35,36,37].

The World Health Organization (WHO) categorizes PH into five distinct categories: (1) pulmonary arterial hypertension (PAH); (2) PH due to left heart disease; (3) PH due to chronic lung disease and/or hypoxia; (4) chronic thromboembolic PH; (5) PH due to unknown causes [38]. PH is characterized by vasoconstriction, pulmonary vascular remodeling, and thrombosis leading to increased pulmonary vascular resistance and right-sided heart failure [39,40,41]. Morbidity and mortality are significant with outcomes closely tied to the status of the right ventricle [42,43,44,45]. While most of the literature discussing the role of 5-HT in the pathogenesis of PH refers to the WHO Group 1 classification, or PAH, it is reasonable to conclude that 5-HT may play a role in the pathogenesis of all groups.

The genetic expression of TPH1 is increased in the lungs and PA-ECs of patients with idiopathic PAH (IPAH) [10]. The pharmacologic and/or genetic depletion of 5-HT synthesis prevents the development of PH in adult mice. When exposed to hypoxia, 5-HT deficient mice (TPH1 knockout mice) demonstrate decreased PA-SMC proliferation, decreased right ventricular hypertrophy, and decreased right ventricular systolic pressures compared to wild-type mice [46,47]. Despite these associations, studies attempting to quantify circulating 5-HT levels in patients with idiopathic PH have yielded conflicting results, perhaps related to the heterogeneity of the disease and difficulty measuring free 5-HT [6].

SERT and 5-HT receptor expression are increased in PH. SERT is highly expressed by lung PA-SMCs and expression is increased in PA-SMCs from patients with IPAH [48]. Pulmonary SERT overexpression is associated with more severe disease in experimental PH, and mice deficient in SERT display attenuated hypoxia-induced pulmonary vascular remodeling and right ventricular hypertrophy compared to wild-type mice [46,49,50,51]. Pulmonary artery expression of the 5-HT 1B receptor is increased in both patients with PAH and mice with experimental PH [24,52]. Further, 5-HT 1B knockout mice display attenuated pulmonary artery vasoconstriction under hypoxic conditions and pharmacologic inhibition of the 1B and 2A receptors prevents the development of experimental PH [21,53,54].

As a result of these findings in the adult population, there is currently a phase 2 multicenter clinical trial (ELEVATE 2) investigating the use of a rodatristat ethyl, a novel TPH1 inhibitor, for the treatment of PH in adults [55]. Blocking the biosynthesis of peripheral 5-HT from tryptophan is hypothesized to decrease circulating 5-HT concentration, thus reducing available SERT substrate, limiting binding to 5-HT receptors, and ultimately ameliorating downstream vasoconstriction and smooth muscle cell proliferation of the pulmonary arteries. This drug has demonstrated efficacy in preclinical models of PH and shown a dose-dependent reduction of the 5-HT metabolite, 5-HIAA, in the plasma and urine of healthy human subjects, though outcomes in humans with PH have yet to be determined [56]. It is clear based on this work that 5-HT plays a well-defined role in the development of adult PH, though relatively little is known about the pathogenesis of PH in the developing lung.

## 3. Serotonin in Fetal Pulmonary Circulation

### 3.1. Serotonin Signaling during Pregnancy and Fetal Development

During pregnancy, 5-HT levels remain constant in the mother but increase in both the placenta and fetus [57]. Consequently, the demand for tryptophan, the essential amino acid that serves as the precursor for 5-HT synthesis, increases to match the needs for fetal growth and development. The fetus is initially dependent on maternal amino acid intake and the placental transport of tryptophan [58]. The placenta serves as an important site of 5-HT synthesis up until mid-gestation, with a subsequent decline throughout the second and third trimesters as the fetus begins independently synthesizing 5-HT [57,59,60]. Human studies have revealed platelet levels of 5-HT are relatively low at birth, but quickly increase and reach concentrations close to adult level by 1 month of life [61].

Disruption in 5-HT signaling during pregnancy has a significant negative impact on fetal growth and development [62,63,64,65,66,67,68,69]. Unsurprisingly, given its known function as a neurotransmitter, 5-HT plays a critical role in fetal brain development and impacts cell migration, proliferation, and maturation [63,70,71,72]. Increased placental 5-HT synthesis may affect fetal forebrain development, leading to long-term behavioral abnormalities such as anxiety [64]. Maternal stress increases tryptophan synthesis and fetal brain concentrations of 5-HT in rats, leading to increased anxiety in the offspring [73]. Meanwhile, impaired placental degradation of tryptophan in humans is associated with an increased risk of schizophrenia, bipolar disorder, and autism [74,75]. While these neuropsychologic consequences have been long appreciated, more recent literature has begun to examine the impact of 5-HT signaling on other biologic processes, including the respiratory and cardiovascular systems.

### 3.2. Serotonin Signaling in the Fetal Lung

In utero, PNECs, which are known to express TPH1, emerge early in gestation during the embryonic stage of human lung development [76]. PNECs are considered to be the most important oxygen and stimuli-sensing epithelial cells in the respiratory system [77]. Despite this important role, they make up just 0.01% of human lung cells [78,79]. 5-HT-containing PNECs, as compared to others (e.g., bombesin), are more prominent during the early stages of development and are present as early as 8 weeks gestation. They are mostly found in larger airways as the epithelium centrifugally differentiates. Preclinical studies in rats have found that 5-HT PNECs increase in number throughout gestation as the fetus nears term and TPH1 expression gradually increases, suggesting 5-HT may play an important role in the neonatal cardiopulmonary transition [60].

SERT expression in the rat lung, amongst other organs (brain, intestine, liver), significantly increases throughout gestation, allowing for improved regulation of 5-HT clearance [60,80]. In the human lung, pulmonary SERT expression begins at 30 weeks gestation, increases to term, and remains postnatally elevated [81]. Studies quantifying 5-HT receptor expression throughout gestation are limited, but have revealed that the pulmonary expression of 5-HT receptors is developmentally regulated, similar to that of SERT. We reported the 5-HT 2A receptor is expressed in the fetal ovine lung and that, compared to the adult ovine lung, the expression of the 5-HT 1B, 2A, and 2B receptors is markedly decreased [82]. Hodge et al. found there is a trend towards increased 5-HT 4 receptor expression in the human fetal lung from 19 days to 19 weeks of gestation [83]. Nikolic et al. analyzed the expression of the 5-HT receptors 5HT 1A, 2A, and 3A across different stages of human lung development from 12 to 40 weeks of gestation [84]. They too found receptor expression was developmentally regulated, with the strongest expression in the epithelium of the proximal airways. 5-HT 1A was also strongly expressed in the smooth muscle cells of the proximal airway, and 5-HT 1A and 2A were expressed by both vascular smooth muscle cells and endothelial cells.

These developmental differences in expression likely have physiologic consequences. While the infusion of 5-HT induces pulmonary vasoconstriction in rabbits of all developmental stages, from fetal through adult life, only 5-HT 2A receptors mediate vasoconstriction in fetal pulmonary vessels, while 5-HT 1B and 1D receptors mediate vasoconstriction in adult vessels [85]. This finding is supported by Goyal et al., who demonstrated the 5-HT 2A receptor is the predominant 5-HT receptor involved in 5-HT-induced vasoconstriction in isolated ovine fetal pulmonary arteries exposed to long-term maternal hypoxia [86]. 5-HT clearly plays a defined role in pulmonary vasoconstriction, but the nuanced mechanisms vary across developmental stages.

## 4. Serotonin in Neonatal Pulmonary Circulation

### 4.1. Neonatal Cardiopulmonary Transition

The definition and classification of PH are the same for children as they are for adults. While there are indeed differences in the etiology, presentation, and outcomes of pediatric PH compared to adult PH, the histopathology and mechanisms for targeted therapies remain similar [87]. However, it is unclear if this same framework should also be applied to infants. In utero, PVR is significantly greater than systemic vascular resistance, allowing for the preferential shunting of oxygenated placental blood to the developing fetus. At birth, ventilation of the newborn lung causes a dramatic decrease in PVR and pulmonary blood flow increases by 8–10 fold to accommodate the postnatal dependence on alveolar gas exchange [88,89]. Pulmonary artery pressures continue to decrease for 2–3 months after birth, a process that takes longer in infants born at higher altitudes. Failure to complete this physiologic transition after birth results in a persistent pulmonary hypertension of the newborn (PPHN), a disease process characterized by sustained elevation in PVR and intracardiac shunting, leading to severe hypoxemia. While the exact incidence and prevalence of PH in the pediatric population are unknown, current registries have begun to examine the etiology associated with pediatric PH. In addition to PPHN, the majority of the remaining cases of PH are caused by idiopathic PAH, PH due to congenital heart disease, or PH due to chronic lung disease of prematurity [90,91,92].

### 4.2. Selective Serotonin Reuptake Inhibitors and Persistent Pulmonary Hypertension of the Newborn

In the early 2000s, several retrospective case-control studies and meta-analyses investigated the association between the maternal use of selective serotonin reuptake inhibitors (SSRI) and the incidence of PPHN. Based on the concerning findings reported by these epidemiologic studies, the United States Food and Drug Administration issued an advisory statement warning against the potential consequences of SSRI use during pregnancy.

SSRIs are a class of medications that block the neuronal reuptake and reabsorption of 5-HT. Approximately 8–10% of pregnant women are prescribed selective SSRIs to treat anxiety and/or depression [93,94,95]. SSRIs bind to SERT expressed by placental syncytiotrophoblasts or cross the placenta and bind to SERT expressed by fetal platelets and organ tissues, resulting in increased local concentrations of 5-HT [96,97,98]. The lung, in addition to the brain, serves as a primary uptake site for SSRIs and may function as a reservoir for antidepressants with a high affinity to SERT [99]. SSRIs are also known to inhibit nitric oxide synthase (NOS) and may impact the concentration of nitric oxide, a potent vasodilator that plays a critical role in the regulation of vascular tone in utero and during postnatal transition [100,101]. Maternal use of SSRIs may compromise uterine/placental vascular perfusion and impact fetal growth. In humans, maternal SSRI use is associated with changes in fetal heart rate and cerebral blood flow, consistent with reductions in placental perfusion and resultant fetal hypoxia [102].

The early epidemiological studies found a six-fold increased risk of PPHN in neonates when exposed to maternal SSRI in pregnancy, specifically during the second and third trimesters [62,103,104,105,106,107,108,109]. However, further examination of this association revealed conflicting data, with an absolute risk difference of 0.6–2 per 1000 live births [95,110,111,112,113,114]. Clinical practice has shown the potential effects of SSRI exposure on respiratory distress are short lived, with no reported deaths. Thus, current obstetric and psychiatric/psychologic practice advises against cessation of SSRI use before or during pregnancy, acknowledging the significant maternal and infant morbidity associated with untreated perinatal depression and anxiety [115].

Despite conflicting evidence regarding the clinical significance of SSRI exposure and PPHN, there is a clear impact of maternal SSRI use on fetomaternal circulation [116,117,118]. Large animal models have shown that acute infusion of the SSRI fluoxetine into pregnant sheep leads to a transient decrease in uterine artery blood flow, fetal PaO2, and oxygen saturation [118]. Similarly, the offspring of rats treated with fluoxetine in late gestation displayed lower postnatal arterial oxygen saturation and PH characterized by increased pulmonary artery medial wall thickness and increased right ventricular mass [119].

Our group utilized the chronically prepared fetal sheep model to study the in vivo hemodynamic effects of SSRIs in the developing lung. We found that brief infusions of the SSRIs sertraline and fluoxetine directly to the fetal ovine lung caused potent and sustained elevation in pulmonary vascular resistance, which could be reversed by the 5-HT 2A receptor antagonist ketanserin [82,120]. Acetylcholine-induced vasodilation persisted after sertraline treatment, suggesting sertraline does not directly impair NOS activity. A similar study in mice revealed sertraline and fluoxetine contribute to in utero constriction of the ductus arteriosus, a well-known mechanism for elevated prenatal pulmonary pressures and PPHN [121]. Based on these mechanisms, it is plausible to conclude the accumulation of lung 5-HT from SSRI exposure could contribute to increased vasoconstriction, smooth muscle cell proliferation, and elevated pulmonary vascular resistance in exposed infants leading to the clinical syndrome known as PPHN.

### 4.3. Serotonin in Neonatal Pulmonary Hypertension

Several decades after the original 5-HT hypothesis was proposed in the literature, a pediatric clinical study quantified 5-HT PNECs in infants who died due to respiratory distress syndrome (RDS) and bronchopulmonary dysplasia (BPD) [122]. Interestingly, the number of 5-HT immunoreactive PNECs was decreased in preterm infants who died from RDS, but significantly increased in those who died from BPD. These results were most pronounced at 2 months of age, when infants with BPD had a 34-fold increase in 5-HT immunoreactive PNECs. The significance of the 5-HT pathway in the process of pulmonary vascular remodeling at birth is further supported by evidence that SERT expression is decreased in infants with alveolar capillary dysplasia with misalignment of pulmonary veins (ACD/MPV) [81]. ACD/MPV is a uniformly lethal condition characterized by severe PPHN. The pulmonary microvasculature of infants with ACD/MPV is markedly depleted of SERT expression, suggesting alterations in 5-HT signaling may contribute to abnormal vascular development and PH. 5-HT metabolism is also increased in pediatric patients with PH due to congenital heart disease [123]. In this population, there is no appreciable difference in the concentration of plasma total and free 5-HT. However, these patients have increased urinary excretion of 5-HIAA, which may be due to increased 5-HT metabolism.

Numerous preclinical studies have demonstrated an important association between 5-HT and PH in both small and large animal models of neonatal PH. 5-HT exacerbates pulmonary artery constriction in a hyperoxic rat model of BPD-induced PH, and pulmonary TPH1 is increased in both murine and ovine models of PH [120,124,125]. In an experimental model of congenital diaphragmatic hernia, a developmental lung disease associated with PPHN and high rates of neonatal morbidity and mortality, the expression of SERT and the 5-HT 2A receptor are upregulated in the pulmonary vasculature, suggesting 5-HT may mediate pulmonary vascular remodeling seen in this disease [126].

Our group studied the hemodynamic effects of 5-HT and 5-HT receptor antagonists in the late-gestation ovine fetus for over a decade [82]. We found that a brief infusion of 5-HT into the pulmonary vasculature increases PVR in a dose-dependent manner. Pharmacologic inhibition of the 5-HT 2A receptor with direct pulmonary infusions of the 5-HT 2A receptor antagonist ketanserin results in pulmonary vasodilation, suggesting endogenous 5-HT contributes to the maintenance of normal pulmonary vascular tone in the ovine fetus via activation of the 5-HT 2A receptor. Ketanserin also prevents exogenous 5-HT-induced pulmonary vasoconstriction. However, pharmacologic inhibition of the 5-HT 1B and 2B receptors does not have this effect. We concluded from this work that the pulmonary vasoconstrictor effects of 5-HT are mediated by the 5-HT 2A receptor and, in part, through rho kinase activation.

We further demonstrated the hemodynamic effects of 5-HT infusion and 5-HT 2A receptor antagonism in a PHHN model of the ovine fetus [120]. As demonstrated in other animal models, prolonged constriction of the ductus arteriosus in fetal sheep leads to elevated pulmonary pressures. This elevation is augmented by 5-HT infusion and ameliorated by 5-HT 2A receptor antagonism. From this we conclude that 5-HT contributes to high PVR in the fetus and speculate that prolonged exposure may impact fetal pulmonary circulation and postnatal transition.

Because both TPH1 and 5-HT 2A receptor expression are increased in experimental PH and the pharmacologic blockade of the 5-HT 2A receptor is protective against the development of experimental PH, we hypothesized that genetic depletion of TPH1 is also protective against PH. Interestingly, this was not the case [127]. Contrary to our hypothesis, circulating and pulmonary 5-HT is decreased in hypoxic wild-type mice compared to the controls. TPH1 knock out mice have a similar degree of hypoxia-induced alveolar simplification and pulmonary vascular remodeling as wild-type mice. Interestingly, the hypoxia-induced increase in right ventricular systolic pressures is appropriately attenuated in TPH1 knock out mice compared to wild-type mice, suggesting local pulmonary 5-HT contributes to pulmonary vascular tone. While the genetic and pharmacologic inhibitions of TPH1 have shown protection in adult models, the mixed findings in neonatal suggest inhibition may have a variable impact in the developing lung.

## 5. Conclusions

PH is a devastating disease that affects adults, children, and infants. Its pathophysiology is driven by a variety of mechanisms that ultimately contribute to disease severity. 5-HT is a potent pulmonary vasoconstrictor and smooth muscle mitogen that has been implicated in the pathogenesis of this disease. 5-HT is both centrally and peripherally synthesized from L-tryptophan through the activity of TPH. Its biologic functions are primarily mediated by SERT and 5-HT receptors. Targeting 5-HT signaling with novel therapeutic agents via these pathways has shown great promise for adults with PH. However, pulmonary SERT and 5-HT receptor expression is developmentally regulated. The limited body of literature describing the role of 5-HT in the developing lung and neonatal PH highlights that this mechanism is quite complicated, and may differ from what is known about 5-HT in adult pulmonary circulation.

PNECs expressing TPH1 are present early in the embryonic phase of development, whereas SERT expression begins later in gestation but gradually rises as the fetus nears term. The 5-HT 2A receptor is the predominant receptor that regulates 5-HT-induced vasoconstriction in the perinatal circulation. Both clinical and preclinical studies suggest that 5-HT signaling contributes to the fetal to neonatal cardiopulmonary transition and that elevated levels of 5-HT may contribute to the development of neonatal PH. Preclinical studies targeting the 5-HT signaling pathway have demonstrated a variable protective effect against 5-HT-mediated pulmonary vasoconstriction, highlighting that we have much to learn about the impact of 5-HT on both lung development and the initiation and progression of PH in newborns. We are hopeful that this field will continue to expand towards improving outcomes for neonates with PH.
